# Clinical spectrum of post-chikungunya rheumatic musculoskeletal disorders and use of disease-modifying antirheumatic drugs to treat the chronic inflammatory entities: 6-year experience from Reunion Island

**DOI:** 10.1186/1471-2334-14-S2-O20

**Published:** 2014-05-23

**Authors:** Emilie Javelle, Anne Ribera, Isabelle Degasne, Catherine Marimoutou, Fabrice Simon

**Affiliations:** 1 Department of Infectious Diseases and Tropical Medicine, Laveran Military Teaching Hospital, Marseille, France; 2 Rheumatology Office, Saint Denis, La Réunion, France; 3 Rheumatology Unit, University Hospital Félix Guyon, Saint Denis, La Réunion, France; 4 Army Center for Epidemiology and Public Health, Marseille, France

## Aim

The re-emerging invalidating chikungunya (CHIK) disease has recently extended to temperate areas. Following the acute febrile polyarthritis, miscellaneous long-lasting rheumatic musculoskeletal disorders (RMSKD) are reported, consistent with chronic inflammatory rheumatisms (CIR) notably rheumatoid arthritis (RA). The post CHIK-infection stage remains a challenge to treat, while the early use of disease-modifying antirheumatic drugs (DMARDs) is recommended in RA. After the 2005-2006 CHIK outbreak in Reunion Island, we retrospectively categorized CHIK-related RSMKD and analyzed the empirical use of DMARDs starting with methotrexate (MTX) to treat the CIR forms.

## Materials and methods

We reviewed medical files of patients referred to rheumatologists in Saint Denis between 12/2005-05/2012 for persisting rheumatic disorders (>4 months) after a proven CHIK infection. We distinguished de novo post-CHIK RMSKD from exacerbation of pre-existing disorders. CIR included: 1) RA defined according 2010 ACR/EULAR criteria, 2) spondylarthropathy (SA) defined according ESSG classification, and 3) undifferentiated polyarthritis (UP) defined as at least three swelling joints. Response to MTX was efficacy versus failure (defined as a need for MTX switch or escalation). Determinants for efficacy were identified using Fisher exact test.

## Results

Among 159 patients included, 122 suffered from de novo RSMKD with 28 presenting chronic pains and 94 fulfilling CIR criteria. RA accounted for 40/92 (12 positive for anti-CCP antibodies); SA for 33/94 (15 with psoriasis); and distal UP for 21/94. 72 of them (including the 31 CIR with joint damages) were treated with MTX (40 RA, 26 SA), reaching efficacy in 54 cases (30 RA, 19 SA, 5 UP) versus failure in 18 cases (10 RA, 7 SA, 1 UP). No severe side event was observed. The group efficacy did not differ with age, sex or type of CIR, but was significantly associated with the early introduction of MTX within the first year of CIR progression (p =0,034).

## Conclusions

Among the wide spectrum of post CHIK RMSKD, CIR should benefit from the early use of MTX. DMARD’s efficacy could bring some clues to investigate the underlying post CHIK-infection disorders.

